# 83. Impact of a Urine Culture Best Practice Advisory on Collection of Urine Cultures and Subsequent Antibiotic Therapy

**DOI:** 10.1093/ofid/ofab466.285

**Published:** 2021-12-04

**Authors:** Logan White, Andrea Dooley-Wood, Hien Nguyen, Aiman Bandali

**Affiliations:** 1 Penn Medicine/Lancaster General Health, Lancaster, Pennsylvania; 2 Atlantic Health System, Summit, New Jersey

## Abstract

**Background:**

In the acute care setting, urinary tract infections (UTIs) may be over diagnosed in up to 40% of cases. In most scenarios, asymptomatic bacteriuria (ASB) is not an indication for antibiotic therapy; inappropriate therapy is associated with a higher incidence of antibiotic-resistant bacteria and adverse drug reactions. Limiting inappropriate collection of urine cultures may decrease unnecessary treatment of ASB. The objective of this study is to assess the impact of a urine culture best practice advisory (BPA) on collection of unnecessary urine cultures.

**Methods:**

This retrospective, observational, single-center study included adult inpatients with an order for urinalysis/urine culture. Those who were pregnant, had a concomitant infection other than UTI and/or were taking antimicrobials for a non-UTI indication, and were undergoing urological procedures were excluded. Duplicate urine culture collections and/or admissions were excluded. Incorporation of a BPA into computerized provider order entry, allowing providers to assess need and document indication for urine culture collection, was implemented on July 2019. The following clinical outcomes were assessed: number of unnecessary urine cultures collected, number of antibiotic treatments, and antibiotic-associated adverse reactions.

**Results:**

Two hundred met criteria for inclusion; 96 in the pre-BPA group (Aug – Oct 2018) and 104 in the post-BPA group (Aug – Oct 2019). Seventy-four (37%) were male and the mean age was 64 and 70 years (p=0.249), respectively. The Charlson Comorbidity Index (CCI) was similar between groups (4 vs. 5, p=0.162) and majority were admitted to a general medical ward (94.5%). Seventy patients (72.9%) in the pre-BPA group and 47 (51.6%) in the post-BPA group had inappropriately ordered urinalysis/urine cultures (OR 0.40; 95% CI 0.22-0.73; p=0.003). Of these patients, 15 (21.4%) and 9 (19.1%) from the pre- and post-BPA groups, respectively, were treated (p=0.077). Among those treated, only two adverse drug reactions were reported.

**Conclusion:**

Implementation of a BPA significantly reduced the number of inappropriate urinalysis/urine culture orders. There was a trend towards decreased antibiotic use for ASB. Future studies are warranted to assess sustainability of these results.

**Disclosures:**

**All Authors**: No reported disclosures

